# Simultaneous Measurement of Neural Spike Recordings and Multi-Photon Calcium Imaging in Neuroblastoma Cells

**DOI:** 10.3390/s121115281

**Published:** 2012-11-08

**Authors:** Suhwan Kim, Unsang Jung, Juyeong Baek, Shinwon Kang, Jeehyun Kim

**Affiliations:** 1 School of Electrical Engineering and Computer Science, Kyungpook National University, 1370 Sankyuk-dong, Buk-gu, Daegu 702-701, Korea; E-Mails: shkim@ee.knu.ac.kr (S.K.); cester@paran.com (U.J.); young02you@empas.com (J.B.); 2 School of Electronics Engineering, College of IT Engineering, Kyungpook National University, 1370 Sankyuk-dong, Buk-gu, Daegu 702-701, Korea

**Keywords:** micro-electrode array, neural spike recording, multi-photon microscope

## Abstract

This paper proposes the design and implementation of a micro-electrode array (MEA) for neuroblastoma cell culturing. It also explains the implementation of a multi-photon microscope (MPM) customized for neuroblastoma cell excitation and imaging under ambient light. Electrical signal and fluorescence images were simultaneously acquired from the neuroblastoma cells on the MEA. MPM calcium images of the cultured neuroblastoma cell on the MEA are presented and also the neural activity was acquired through the MEA recording. A calcium green-1 (CG-1) dextran conjugate of 10,000 D molecular weight was used in this experiment for calcium imaging. This study also evaluated the calcium oscillations and neural spike recording of neuroblastoma cells in an epileptic condition. Based on our observation of neural spikes in neuroblastoma cells with our proposed imaging modality, we report that neuroblastoma cells can be an important model for epileptic activity studies.

## 1. Introduction

In recent times, neuroscience has received much attention as important research field for understanding the connections between neuron cells and reaction mechanisms. These kinds of research have been focused on recording electrical signals from living brains or cells, called neural spikes, for studying the activity of the brain or neuron cells. A component of instrumentation development for cell study is also included in this research [1,2]. Many researchers have demonstrated good results in the field that measures extracellular signals from individual neurons for over 50 years [3–5]. Also, it became possible to measure reaction signals from tens to hundreds of neurons [6], but in all this research, there exists a limitation that the neurons cannot be observed while acquiring signals from them. To overcome this limitation, studies using two-photon or multi-photon microscopy as imaging tools have been underway since the late 1990s [7–9]. Such non-linear microscopies are becoming standard tools for defining molecular mechanisms in the field of cell-based engineering and bio-medical research. Most of all, multi-photon microscope (MPM) is a type of laser-scanning microscope that excites a fluorescent material to a thin raster-scanned plane using a ‘non-linear’ excitation state [10]. Ten years after the first report on MPM, it has been applied to various imaging fields, and nowadays it has become an imaging modality used to observe thick tissues or living animals.

Barbashov *et al.* and Alexander *et al.* reported the multiphoton excitation of nerve cells using femtosecond laser radiation [11,12]. Ryan *et al.* showed their experimental results with signals recorded from the visual cortex using a microelectrode array and a single electrode [13]. Because just using the MPM system or extracellular recording is confined to imaging or electrical signal recording, a complex system which can perform simultaneous measurements of the neuron images and the electrical signals could improve the reliability of experimental results. If a planar multi-electrode array is applied to culturing neuron cells and compatible with an imaging modality, simultaneous acquisition of the neuron images and electrical signals could be done. Also, by using a specific calcium dye, the system can have additional merit if we can analyze the activity of calcium channels from cultured neuron cells. A multi-signal acquisition system that performs a simultaneous measurement of electrical signals, fluorescence images and changes would have an important role in neuroscience. Shew *et al.* already reported about a system which performs the simultaneous measurement of neural activity using a complex multi-electrode array and a two-photon microscope [14]. They focused on the large-scale field potential signals to single-neuron activity in small scale-group cells from rat brain slices.

For differentiated experimental results, our study focused on not only a simultaneous measurement system using a micro-electrode array of simple structure, but also on recording neural spikes from cultured neuroblastoma cells under epileptic conditions. Recently, neuroblastoma cells have been considered as an attractive model for the study of human neurological and prion diseases, and intensively used as a model system in different areas. Among those areas, differentiation of neuroblastoma cells, receptor-mediated ion current, and glutamate-induced physiological responses are being actively investigated. The reason for the interest in neuroblastoma cells is that they have a faster growth rate than other cells on neural origin with a few another advantages. This model has not been reported as far as the authors know.

In order to develop a multi-signal acquisition system, this paper describes the design and implementation of the fabricated MEA for neuroblastoma cell culturing and signal recording. It also explains the MPM system used for the calcium imaging of the cultured neuroblastoma cells. The developed MPM system is miniaturized, so the whole system can be mounted onto a medical cart through downsizing of a fs Ti-Sapphire mode-locked laser source. To achieve simultaneous electrical signal acquisition, fluorescence images and changes from the cultured neuroblastoma cells, we collected all measurands with a data acquisition (DAQ) board and observed all signals through the a program written with the developed software. Using the experimental results based on the developed system, we could explain the neuroblastoma cell based epileptic model and analysis of calcium oscillation and neural spikes in seizure events. This could potentially be a basic model to understand the epileptic process in neuroblastoma cells. These kinds of system and results play an important role to better understand the neural signaling of neural networks.

## 2. Materials and Methods

### 2.1. Microelectrode Array Design

**Figure 1.**
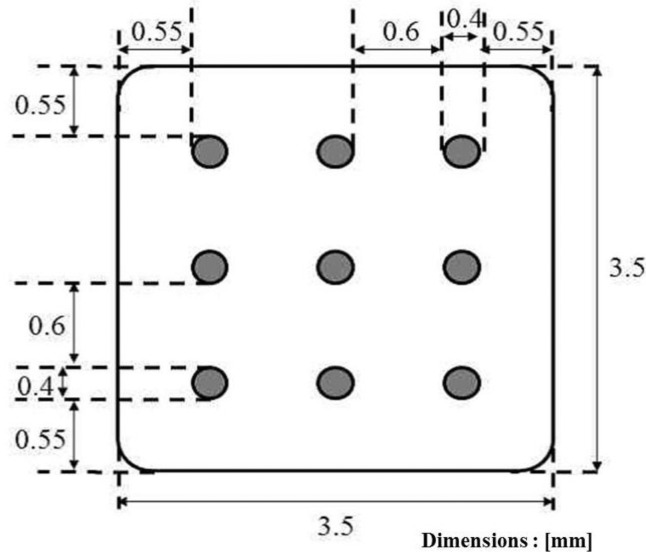
Dimensions of the microelectrode array.

A flexible printed circuit board (PCB) was used for the electrode design. Polyimide is the most common substrate material for flexible PCBs because it possesses good biocompatibility with neuron cells and allows the neurons to grow on its surface. The MEA is often covered with copper as a conductive layer, and it was coated with gold to enhance the biocompatibility. The dimensions of the MEA are 3.5 mm × 3.5 mm. The diameter of each electrode element was 400 μm. The connecters of the electrodes are located at 2.5 mm away from the electrode. The MEA has in total nine electrodes in a 3 × 3 arrangement as shown in Figure 1. All the dimensions shown in figure are in mm.

### 2.2. Neuroblastoma Cell Culture

Neuroblastoma N2A cells were purchased from the American Type Culture Collection (ATCC, CCl-131) and cultured in Dulbecco's modified Eagle medium (DMEM) supplemented with 10% Fetal Bovine Serum (FBS) in a CO_2_ incubator (Forma 3111, ThermoFisher Scientific, Waltham, MA, USA) supplied with 5% CO_2_ at 37 °C. Confluent cells were digested with 0.25% trypsin, followed by centrifugation (1,500 rpm for 5 min) to harvest the cells. Subsequently, single cell suspensions were used to calculate the cell number with a hemocytometer. For each sample, 1.0 × 10^6^ cells were added to the medium and cultured for 24 hour. First we fixed the MEA in a culture dish then overnight UV sterilization was performed on the MEA before the experiment. Cells (1.0 × 10^6^) were added on the MEA and then incubated for 3 hours in 24-well cell culture plates. After 3 hour, DMEM was added for proper cultivation of cells. Then again it was placed in the incubator for 6 days.

### 2.3. Neural Spikes Recording and Multi-Photon Microscope System for Calcium Imaging

**Figure 2.**
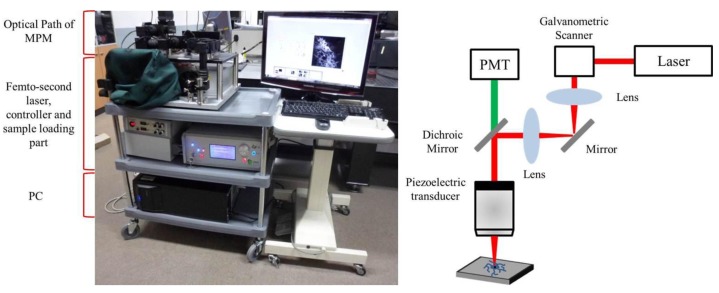
A photograph and the structure of the MPM system.

The developed MPM system for simultaneous detection of the calcium dye and the electrical signals is miniaturized by two methods. First, we decreased the volume of a fs Ti-sapphire mode-locked laser. We used the small semiconductor laser as a pump laser source, and put it in front of a laser cavity system. The sizes of all the optics used in the laser cavity system are minimized through optimization, so the volume of the laser cavity, including the pump source, is just 400 × 250 × 110 mm^3^. We adopted an air cooling method with a fan and an aluminium (Al) heat sink instead of a liquid circulation cooling method. The Al heat sink was located under the laser cavity for dissipating the heat. Secondly, an optical path for MPM is constructed with a two-layer system. The beam from the end of the laser cavity was reflected by a mirror and transferred to the upper layer. Expansion and scanning procedure are implemented in the upper layer to decrease the volume of the MPM system. The structure of the implemented MPM system is shown in Figure 2. The average power of the laser was above 600 mW when it was mode-locked and the tuning range of the center wavelength was 740∼840 nm, and it is variable for other applications.

For analyzing the calcium dynamics of cultured neuroblastoma cells, we measured the mean pixel value of the imaging area for each frame from the PMT signal. The fluorescence change was defined as a delta function of *ΔF/F(t)* = (F_0_ − F(t))/F_0_, expressed in %, where F_0_ is the average fluorescence intensity in the first four frames of the imaging area from the start of recording, F(t) is the fluorescence intensity at given time [15]. The fluorescence image was simultaneously recorded with the electrical signals and the fluorescence change was directly calculated from the image and shown on the main screen of the developed LabVIEW software interface.

The electrical signals from the cultured neuroblastoma cells on the implemented MEA are directly entered into the AC amplifier (Model 3600 16 Channel Extracellular Amplifier, A-M Systems, Carlsborg, WA, USA) and the amplifier is connected to the DAQ board (NI PCIe-6321, National Instruments, Austin, TX, USA). We utilized the MEA instead of a needle type probe for exact measurement of the designated imaging area. This can increase the reliability of the simultaneous measurements of calcium imaging, fluorescence change and electrical signals. The cut-off signal of the low-pass filter was 100 Hz and the high-pass filter was 10 kHz. The amplification performed by the main amplifier circuit is 2 k and a notch filter was activated.

**Figure 3.**
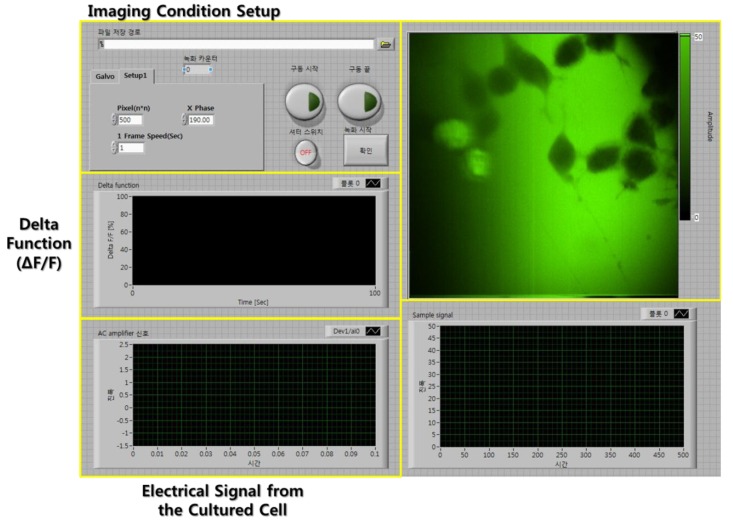
The main screen of the neural spike recording and fluorescence imaging program based on the LabVIEW.

We wrote software compatible with a galvanometric scanning system and the photon multiplier tube (PMT; H7521-40, Hamamatsu, Bridgewater, NJ, USA) based on the LabVIEW software. As the order for driving the program, first, we set a range of two-axis galvanometric scanning system and a pixel number of the images. From that, signals are generated for the two-axis galvanometric scanning system using the scan range value and the pixel number. Then, a start trigger is generated for simultaneous driving of the two-axis scanning and DAQ from the PMT. Receiving the start trigger, a reference trigger for two-axis scanning and pulse counting at the PMT is generated, then the pulse counting begins. During the image processing, the signal acquisition from the cultured cells and fluorescence changes, which are expressed as percentage relative to baseline (*ΔF/F*), are also calculated. Figure 3 shows the main screen of the developed MPM software interface.

### 2.4. Induction of the Epileptiform Event

We took the 7 day old cells from the incubator and removed the media. Then we put the 5 mL Krebs solution into the cells and put them back in the incubator for 2 hours. Then we added 4-aminopyrodine (AP) with a 100 μM concentration in the Krebs solution and again incubated them for 30–50 min.

## 3. Results and Discussion

### 3.1. Neural Spikes Recording

**Figure 4.**
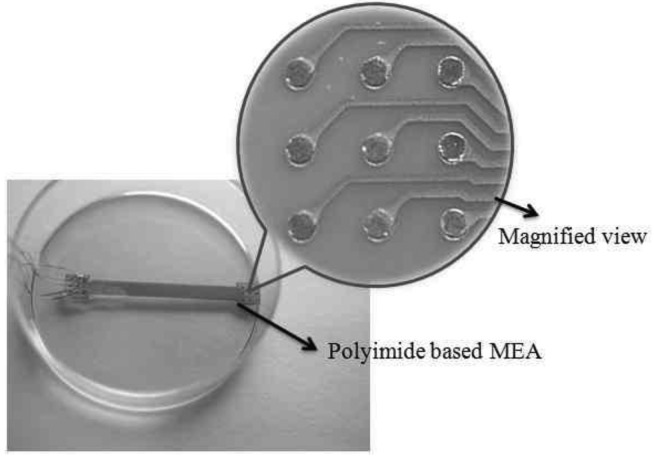
The implemented micro-electrode array.

**Figure 5.**
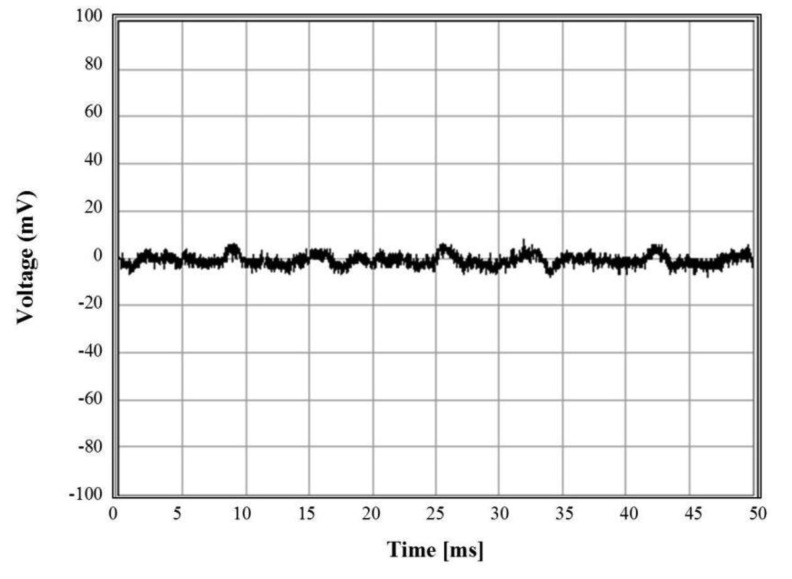
The neural spikes of neuroblastoma cells cultured for 7 days.

Figure 4 shows the implemented MEA on which the neuroblastoma cells are cultured to generate the neural spikes. The neural signal recording has been performed using the polyimide based MEA. For the signal recording, the neuroblastoma cells were cultured on the MEA for 7 days allowing the cells to attach to the MEA surface. We measured the electrical signal from the MEA for 50 ms and Figure 5 shows the neural spike recording result, where the maximum amplitude of the recorded signal was about 5 mV.

**Figure 6.**
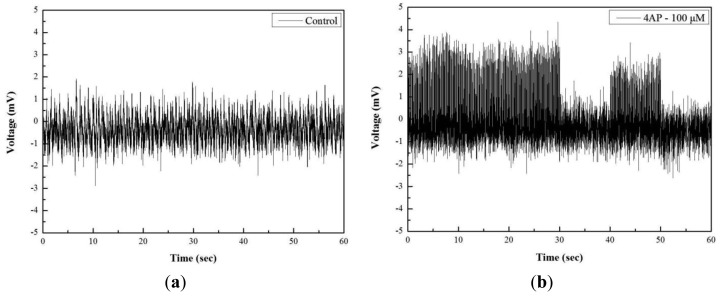
Extracellular neural spikes recording; (**a**) control cells; (**b**) 30 min after injection of 4-AP (100 μM).

Figure 6 shows other neural spikes from the neuroblastoma cells. Figure 6(a) shows the control signal before producing the epilepsy in the cells. From the figure it was observed that neural spikes have low magnitude and frequency. Figure 6(b) shows the neural spike date acquired at 30 min after injecting the 4-AP (100 μM). It was observed that the occurring frequency and the amplitude of the spikes are increased, which indicates the onset of the epileptic seizure events. The amplitude of the signal was increased to twice of the control signal from 1.5 mV to 4 mV. This increment is caused by the ability of the 4-AP inhibition of voltage-gated K+ channels [16], and 4-AP also increases the release of the neurotransmitters such as glutamate.

### 3.2. Calcium Imaging and Delta Function

For the calcium imaging, the cultured cells were stained with a Calcium Green-1 (CG-1) dextran conjugate indicator of 10,000 D molecular weight. We added 3 μL of CG-1 to the culture medium with a micro-pipette and waited for 5 minutes until it diffused to the designated area. Then the dye-loaded cells were visualized using the developed MPM system equipped with a water immersion lens (C-Apochromat series 40×, NA 1.2, working distance 0.28 mm/cover glass 0.17 mm, Zeiss, Oberkochen, Germany). We fixed the wavelength of the fs laser source at 800 nm, so the resolution of the developed MPM system was 0.33 μm. Multi-photon excited fluorescence was separated by a dichroic mirror of 600 nm.

**Figure 7.**
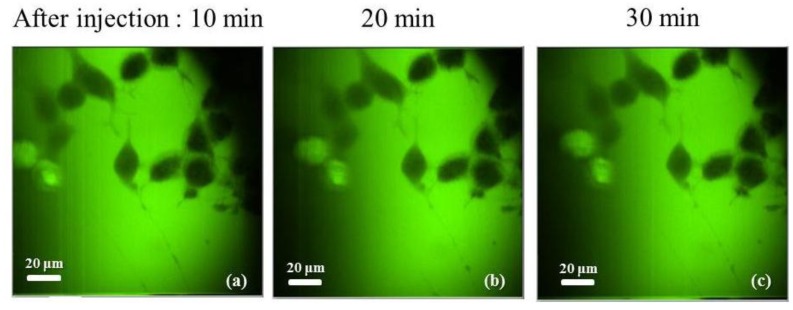
Consecutive fluorescence images taken 10, 20, 30 min after injection of 3 μL of CG-1 to cultured neuroblastoma cells.

**Figure 8.**
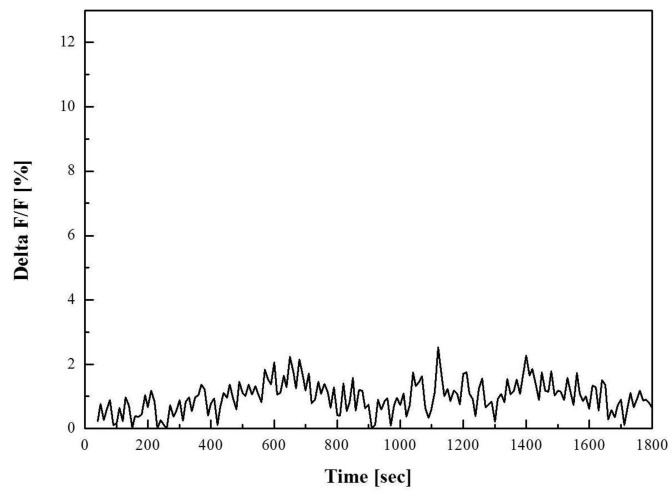
The delta functions of cultured neuroblastoma cells taken during 30 min after injection of 3 μL of CG-1.

First, for confirming the stability level of fluorescence within stain cells, we took images of the neuroblastoma cells outside of the MEA and show these results in Figure 7. Until 30 min, there is no change of fluorescence level in the time course after dye injection as shown in the figure. It can be also proven with the delta function shown in Figure 8. The change of fluorescence was under 2%. We speculate that the variation was caused from the noise of the galvanometric mirror and DAQ board.

**Figure 9.**
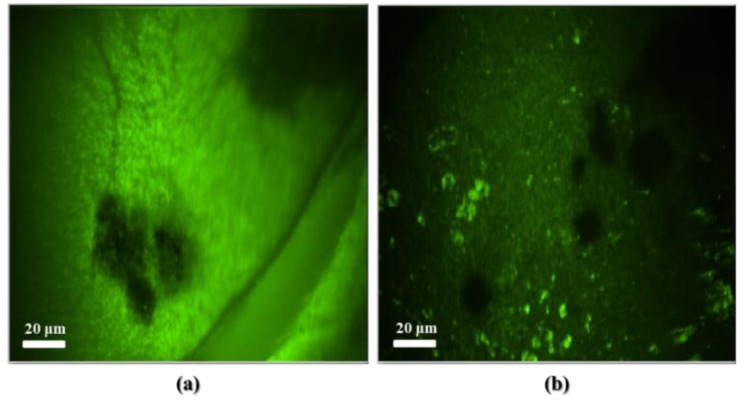
Fluorescence images of neuroblastoma cells on the PCB MEA of (**a**) side part and (**b**) center part.

Then the cultured cells on the PCB MEA were observed after 10 min from the injection and the result are shown in Figure 9. Though many cells are not attached on all electrodes, we could clearly observe cells at all electrodes. Figure 9(a) shows the cell body at the side part of MEA and Figure 9(b) shows those at the center part of MEA. We could clearly see the dendrites from the cells of side part, but not from the cells of center parts because of contrast difference.

**Figure 10.**
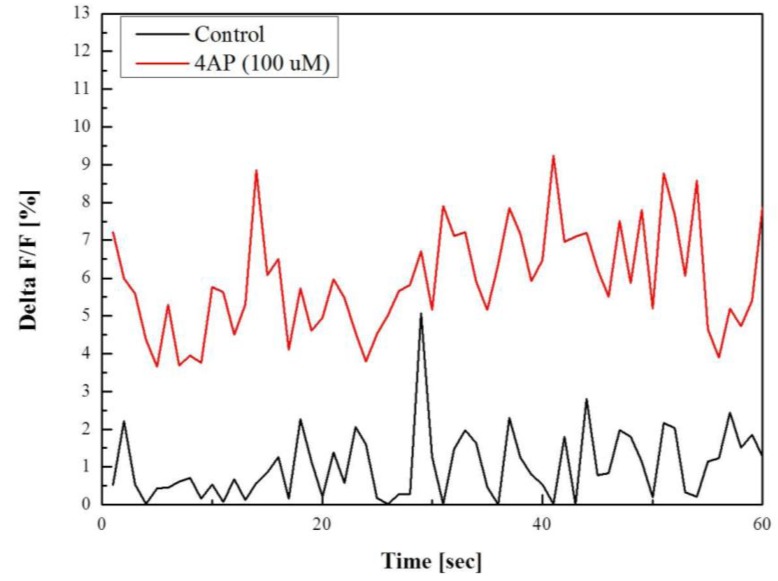
The calcium oscillation in neuroblastoma cells: control cells (black line) and 30 min after the injection of the 4-AP with 100 μM (red line).

The calcium oscillation can be observed by plotting the difference in the electrically excited fluorescent signal from the dormant fluorescent signal as defined in the calcium imaging with MPM session. Figure 10 shows the delta function of the control cells and 30 min after the injection of the 4-AP with 100 μM. The graphs indicate the calcium signal changed after the injection. The red line shows the higher amplitude in the delta function as the effect according to the injection of 4-AP and calcium ion concentration resulting in higher neural activities. The results reflect the onset of an epileptic condition in the neuroblastoma cells because the 4-AP is a K+ channel blocker which is widely used to generate the epilepsy in the hippocampus, cortical neurons, and astrocytes.

After injection of the 4-AP these neuroblastoma cells start producing rapid neural spikes. From the results, extensive neural spikes have been observed from the neuroblastoma cells, which indicates the generation of the seizure like events in the cells.

## 4. Conclusions

In this paper, the system of calcium imaging, fluorescence and electrical signal measurement for cultured neuroblastoma cells was described. All measurements were performed simultaneously for exact signal acquisition from a designated imaging area. The developed MPM system provided stable fluorescence detection from the cultured neuroblastoma cells on the MEA over 30 min. Moreover, the cells on the electrode could be detected clearly, not just on the flat culture dish. Simultaneously, the electrical signals and fluorescence change could be stably acquired. From those measurements, we proved suitability of the signals and stability of developed the MPM and the measurement system.

In a field of medical and cell experiment application, multi-signal acquisition is necessary for diverse signal analysis. Based on these results, our measurement system can be applied not only for cell experiments in a laboratory but also in medical applications because the system is portable enough to load on a movable cart. In the future, we are going to apply this system to various calcium dyes and other kinds of neuron cells for observing calcium oscillation and acquiring the signals simultaneously.
